# Psychiatrization in mental health care: The emergency department

**DOI:** 10.3389/fsoc.2022.793836

**Published:** 2022-09-23

**Authors:** Timo Beeker

**Affiliations:** Department of Psychiatry and Psychotherapy, Brandenburg Medical School Theodor Fontane, Immanuel Klinik Rüdersdorf, Rüdersdorf, Germany

**Keywords:** psychiatrization, emergency care, case study, transdisciplinary research, psychiatric epidemiology, medicalization, overdiagnosis, health system research

## Abstract

**Background:**

In the light of high incidences of diagnosed mental disorders and the growing utilization of mental healthcare services, a progressing psychiatrization of society has been hypothesized as the underlying dynamic of these developments. Mental healthcare institutions, such as psychiatric hospitals, may play a decisive role in this. However, there is a scarcity of research into how psychiatrization emerges in hospital settings. This paper explores whether the emergency department (ED) can be considered as a site where psychiatrization happens, becomes observable, and which factors in the context of the ED may be its potential drivers.

**Methods:**

Two cases as encountered in an interdisciplinary ED will be presented in the following in an anonymized way. Although the cases originate from individual consultations, they can be considered as prototypical. The cases were collected and discussed using the method of interactive interviewing. The results will be analyzed against the backdrop of current theoretic concepts of psychiatrization.

**Findings:**

The ED can be seen as an important area of contact between society and psychiatry. Decisions whether to label a certain condition as a “mental disorder” and to therefore initiate psychiatric treatment, or not, can be highly difficult, especially in cases where the (health) concerns are rather moderate, and clearly associated with common life problems. Psychiatrists' decisions may be largely influenced in favor of psychiatrization by a wide array of disciplinary, institutional, interpersonal, personal, cultural, and social factors.

**Conclusions:**

The ED appears to be a promising field for research into the mechanisms and motives through which psychiatrization may emerge in mental healthcare settings. Psychiatrists in the ED work within a complex sphere of top-down and bottom-up drivers of psychiatrization. Encounters in the ED can be an important step toward adequate support for many individuals, but they also risk becoming the starting point of psychiatrization by interpreting certain problems through the psychiatric gaze, which may induce diagnoses of questionable validity and treatment of little use.

## Introduction and state of research

### Psychiatrization

On a global scale, there have been claims of consistently high or even rising incidences of mental disorders over the last decades (World Health Organization, 2019), resulting in an increasing financial burden on the global economy (Chisholm et al., 2016; World Health Organization, 2016). Survey-based epidemiological studies suggest a lifetime-prevalence of nearly 50% for a mental disorder among the US-population (Kessler et al., 2005; NIMH, 2019), while a meta-analysis across 63 countries identified an average 12-month prevalence of 17.6% for common mental disorders (Steel et al., 2014). These findings resonate well with similar or even higher numbers that are popularized by various mental health advocacy groups and awareness campaigns (MIND, 2021; NAMI, 2021). Currently, there is also widespread concern that the incidences of mental disorders may rise even further due to the COVID-19 pandemic (Hossain et al., 2020; Nearchou et al., 2020; Torales et al., 2020; Kola et al., 2021).

These high incidences are paralleled by a steadily growing utilization of in- and out-patient mental health services, which regularly entail the prescription of psychotropic medication (Lipson et al., 2019; Olfson et al., 2019). While prescription-rates for antidepressants more than doubled in many OECD countries from 2000 to 2015 (OECD, 2020), one in six US-adults is estimated to be on psychotropic medication over the course of a year (Moore and Mattison, 2017). Explanations for these developments are diverse. On the one hand, improvements in recognition and destigmatization of mental disorders are speculated to be causal (Mojtabai, 2010; Richter and Berger, 2013; Mars et al., 2017) as well as deteriorating working and living conditions (Ehrenberg, 1998; Eckersley, 2005; Dittmar et al., 2014; Rosa, 2015). On the other hand, overdiagnosis (Moynihan et al., 2012; Frances, 2013) and flaws in epidemiologic methodology (Horwitz and Wakefield, 2006; Jorm, 2006; Brhlikova et al., 2011) may also contribute to what appears to be a global mental health crisis.

**Figure 1:**
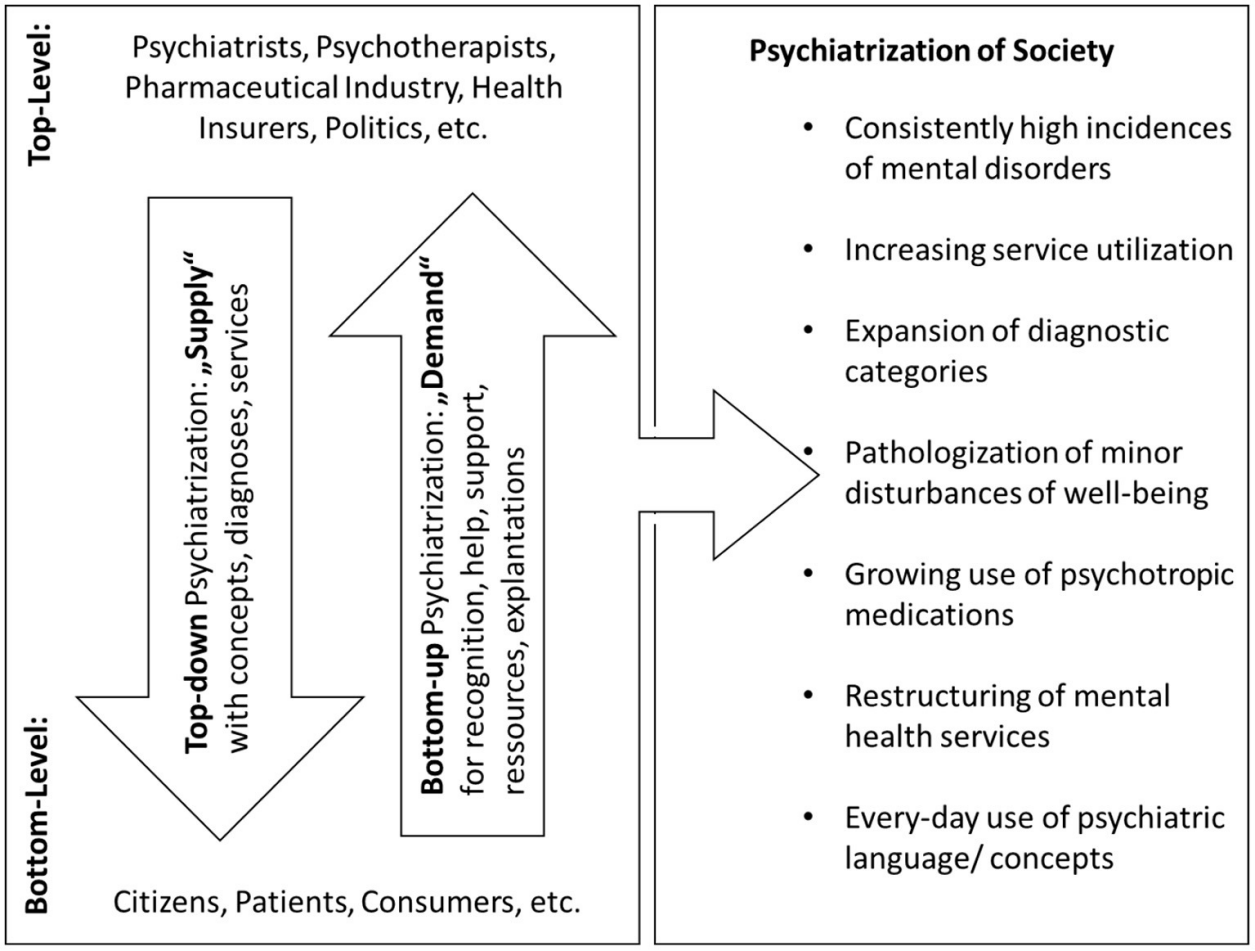
Top-down and bottom-up psychiatrization. Main protagonists and vectors of psychiatrization consisting of heterogeneous sub-processes, of which the most important are listed on the right side of the figure. First published in Beeker et al. (2021a).

In Beeker et al. (2021a), it has been suggested to understand the high, or rising incidences and the growing utilization of mental health services as different parts of a higher-order sociocultural process, which could be described as a *psychiatrization of society*. Psychiatrization is defined there as “a *complex process of interaction between individuals, society and psychiatry* through which psychiatric institutions, knowledge, and practices affect an increasing number of people, shape more and more areas of life, and further psychiatry's importance in society as a whole” (p. 3). Psychiatrization, thus, is conceived of as dynamic, heterogeneous, and as consisting of various sub-processes. The latter may comprise material as well as ideological aspects (see Figure 1).

The effects of psychiatrization are deeply ambivalent. On the one hand, some individuals or groups might benefit from lower-threshold access to an expanding mental healthcare system. Especially in underserved areas, the installation or the strengthening of facilities providing mental healthcare may first address existing unmet need and help close what has been referred to as “treatment gap” (Lancet Global Mental Health Group et al., 2007; Thornicroft et al., 2017). The more widespread provision of care and easier access to it may also be important steps toward a normalization of seeking professional help for what is widely perceived as mental disorders. That could contribute to lowering the remaining high pressures through stigma on people suffering from different kinds of mental distress (Thornicroft et al., 2009; Henderson et al., 2014). On the other hand, there is growing concern about the potential harms of psychiatrization. To individuals, psychiatrization may be detrimental through overtreatment and overdiagnosis (Kirsch et al., 2008; Turner et al., 2008; Whitaker, 2010; Read et al., 2014), or the psychological burden of being labeled (Livingston and Boyd, 2010; Chang and Bassman, 2019). From a public health perspective, psychiatrization risks exploding healthcare costs and widespread inverse care (Hart, 1971; Miller et al., 1988; Wang et al., 2007). In society as a whole, psychiatrization may further narrow down the scope of what is perceived as “normal” and encourage medical solutions for social and political problems (Behrouzan, 2016; Brinkmann, 2016; Davies, 2017; Klein and Mills, 2017).

One core feature of psychiatrization is its strong drive towards expansion. On a structural level, this may happen through the steady growth of psychiatric infrastructures or by changes in diagnostic practices (Rose, 2006; Batstra and Frances, 2012a; Cosgrove and Whitaker, 2015; Paris, 2015). These changes may be accompanied by more subtle transformations in discourse and public opinion, e.g., when psychiatric concepts become widely popularized and negative experiences are increasingly perceived through the psychiatric lens (Furedi, 2004; Brinkmann, 2016; Haslam, 2016). On the individual level, psychiatry may expand when people seeking help for common life issues, or mere individual variation are turned into psychiatric patients by being diagnosed and treated as mentally ill. This kind of low-threshold psychiatrization risks initiating avoidable patient careers by obscuring individual or life problems with psychiatric concepts. This may encourage individual identification with psychiatric labels, a weakening of self-efficacy, and, thus, ultimately create dependency on the mental health services (Rose, 2003; Martin, 2007; von Peter, 2013; Haslam and Kvaale, 2015).

In the medical field, research from different disciplines has shed light on various developments that bear relevance as the origins, mechanisms, or effects of psychiatrization, among them overtreatment, overdiagnosis, inflated epidemiological data, drug-safety, and the rising prescription rates of psychotropic medication (Castner et al., 2000; Horwitz and Wakefield, 2006; Faber et al., 2012; Moynihan et al., 2012; Read et al., 2014). In this context, several measures which aim at countering some of the negative effects of psychiatrization have been suggested or applied on a relatively small scale, such as introducing stepped diagnosis (Batstra and Frances, 2012c), implementing open dialogue as a less psychiatrizing means of psychosocial support (von Peter et al., 2021), advocating alternative frameworks to psychiatric diagnosis (Baumgardt and Weinmann, 2022), limiting the influence of psychiatric corporate interest and pharmaceutical companies (Frances, 2013; Cosgrove and Whitaker, 2015) or, with a growing importance, fostering user-involvement in research and care (Gillard et al., 2010; Wright and Kongats, 2018; Beeker et al., 2021b). Nevertheless, a far wider array of aspects of psychiatrization has been described in non-medical disciplines, such as anthropology, critical psychology, sociology or Mad Studies, using different theoretical frameworks, methodologies, and terminologies (LeFrançois et al., 2013; Behrouzan, 2016; Jain and Orr, 2016; Russo and Sweeney, 2017). Research on psychiatrization heavily draws on the existing body of scientific research on medicalization (Zola, 1972; Illich, 1974; Conrad, 1992, 2005, 2007), biomedicalization (Clarke et al., 2003), pharmaceuticalization (Fox and Ward, 2008; Abraham, 2010; Jenkins, 2011), therapeutization (Furedi, 2004; Sommers and Satel, 2005), and psychologization (De Vos, 2010; Gordo Lopez and De Vos, 2010; Haslam, 2016). Seminal works in this broader context are, among others, those of the philosopher of science Ian Hacking on how psychiatric classifications evolve while circulating in “feedback-loops” between psychiatry and society and their power of “bringing into being” different kinds of people (Hacking, 1985, 1995a,b, 1998, 1999). Many arguments of those different strains of thought are still fueled by the radical skepticism toward psychiatry expressed in the classic anti-psychiatric literature of the 1960s and 70s (Goffman, 1961; Foucault, 1965; Laing, 1965; Cooper, 1967). Authors like Thomas Szasz famously challenged the idea that the behaviors or the emotions of human beings, however aberrant or unusual they may be, can be meaningfully construed as “mental illness” in the same sense as a somatic disease can be conceptualized (Szasz, 1974). Instead, the seemingly scientific disease categories of psychiatry would rather rely on moral judgment and social convention than on any kind of physiological basis. By casting fundamental doubts on the medical nature of psychiatric conditions, anti-psychiatric authors also raised the question if psychiatry can rightfully claim to be a medical specialty at all.

As a potential starting point for transdisciplinary inquiry into psychiatrization, a comprehensive model (see Figure 1) has been suggested in Beeker et al. (2021a). The relevant protagonists of psychiatrization have been classified into agents on the top- and the bottom-level, the latter consisting of “laypeople” without professional ties to the mental healthcare system. Within this model, drivers of psychiatrization can be imagined as vectors running either from top to down or vice versa, which can also involve the looping effects theorized by Hacking (1985, 1995b). On a large scale, top-down psychiatrization may be driven by diverse factors such as political decisions, interests of psychiatric professionals' organizations, scientists promoting their area of expertise, or financial incentives (Scott, 2006; Conrad, 2013; Cosgrove and Whitaker, 2015; Horwitz, 2015). Motives for bottom-up psychiatrization may be the “needs and desires of patients, proto-patients and consumers” (Beeker et al., 2021a, p. 5).

### The emergency department as research field

There is a need for research on where exactly, how, and for which reasons psychiatrization emerges from mental health care. Against this background, the interactions between psychiatrists and people in need of help, which are taking place within the institutions of clinical psychiatry, are of particular interest. In many countries, psychiatric hospitals or general hospitals with psychiatric divisions are a central pillar of the mental healthcare system. In Germany, more than half of the practicing psychiatric specialists work in hospitals (DGPPN, 2021), making them an important locus for the research on psychiatrization. Within psychiatric hospitals, many *first contacts* between psychiatry as an institution and people in mental distress take place in the emergency department (ED), where clinical psychiatrists encounter people who may often be in the middle of an acute situation of crisis, which requires an interpretation that may entail psychiatric diagnosis and treatment as well—or not. From a sociological point of view, the decision whether a medical diagnosis is conferred may have important implications, as it determines if an individual obtains the entitlements and obligations associated with what Talcott Parsons coined as the “sick role” (Parsons, 1951). The ED might, thus, be a particularly interesting site for the inquiry into the origins, motives, mechanisms, and effects of psychiatrization, and into how these become tangible in the everyday work of clinical psychiatrists.

In multidisciplinary EDs, different medical specialties provide treatment to a broad spectrum of illnesses and injuries. They are the common entry point for patients in need of immediate care at hospitals in Germany and many other countries (Roppolo, 2007; Wyatt et al., 2012). Patients usually come to the ED without an appointment. They are either brought in by ambulance or arrive by their own means. Typically, the identification of the primary concern, a first assessment of the severity of the case, and the assignment to the medical specialty in charge (“triage”) is performed by nursing professionals (Wyatt et al., 2012, p. 7). If hospitals dispose of a psychiatric department, the triage will also preselect patients for referral to the psychiatrist on duty. Broadly speaking, the main task of the psychiatric specialist is to recognize and specify mental disorders in accordance with the standards of psychiatric classification, and, if appropriate, to initiate treatment.

There is a variety of research from the social sciences on several aspects of the ED as “a complex space that can be interpreted on individual, societal and systemic levels” (Grace, 2020, p. 875). Scholars generally acknowledge the centrality of the ED in the organization of hospitals (Vosk and Milofsky, 2002c; Hillman, 2016; Grace, 2020). In this context, the ED serves as “gateway to higher levels of medical care” (Grace, 2020, p. 876). Organizational sociological perspectives often stress the fact that medical care in the ED is delivered rather by multidisciplinary teams than by individual specialists, making the ED a promising ground for the inquiry into interprofessional interactions and social hierarchies (Vosk and Milofsky, 2002a,b; Grace, 2020). Another branch of research focuses on gatekeeping-processes, which have a long tradition of being perceived as value-laden or economically driven exclusionary practices which can serve as barriers denying access to medical care to vulnerable groups (Jeffery, 1979; Dingwall and Murray, 1983; Hughes, 1989; Vassy, 2001; Hillman, 2014). The ED may, thus, contribute to and perpetuate basic health inequalities. However, there are also more ambivalent findings in this regard. Dodier and Camus (1998) characterize the ED's functioning as situated within a tension between the two poles of “openness” to spontaneous and heterogenous demands for medical care and “specialization”. This points to the ED's task of selecting patients, who are eligible for immediate care, and of referring them to the responsible medical specialty. In a similar vein, Hillman's (2016) ethnographic study of an NHS hospital reveals how staff at the ED copes with the increasing tensions between their own moral commitment to good care and institutional concerns about resource rationalization and accountability. In a similar vein, Buchbinder (2017) challenges the traditionally negative connotations of gatekeeping and advocates a more balanced view, which does not narrow down its functioning to restrictive, exclusionary practices. More importantly, gatekeeping in the ED may rather facilitate the provision of appropriate medical care by diverting patients to alternative, better fitting sites of treatment or non-medical support, which often aligns well with' the genuine interests of the patients (Buchbinder, 2017).

In the following passages, two cases of psychiatric consultations in the ED of a general hospital will be presented. They will serve as material for exploring how psychiatrization may occur in psychiatric hospital settings as a first step toward the realization of broader empirical studies with larger samples and a wider array of methods. The exploration and analysis will be guided by following preliminary research questions: Is the ED, as an area of contact between psychiatry and society, a place where psychiatrization may emerge in an observable way? If so, which aspects of psychiatrization can be found there? Which top-down and bottom-up drivers of psychiatrization may become tangible in the ED? What can we learn about psychiatrization by analyzing the interactions between ordinary psychiatrists and patients in this specific setting? And, ultimately, how do these findings relate to previous conceptualizations of psychiatrization?

## Methods and material

### Approach and position of the author

Since psychiatrization itself is an interdisciplinary research object in between medicine and the social sciences, this case study posits itself in the tradition of two different methodological approaches with regard to the selection and analysis of individual cases. In the medical tradition, case studies or case reports usually present particular cases in which medical professionals had to deal with extraordinary challenges concerning diagnosis and treatment of a patient (Carleton and Webb, 2012). As such, case studies do not aim at generating statistically significant outcomes, but at an in-depth understanding of a special phenomenon or situation. Detailing clinical considerations and decisions, a case study is understood to have a double function: It serves as educational material for practitioners as well as a prospective first step toward the design of specific clinical studies with the aim of further investigating the phenomenon described (Nissen and Wynn, 2014). Case studies are typically written by the medical experts who themselves were responsible for the management of the particular case. The author's double role as the practitioner who handled the case and thereby actively contributed to the production of the material that she later describes and analyzes as researcher is usually not problematized. Rather, such case studies are valued for this kind of ex-post self-reflectivity, which may help to improve the provision of care in similar cases in the future (Solomon, 2006; Budgell, 2008; Carleton and Webb, 2012).

In the social sciences, case studies on medical topics are usually far more complex, refer to larger sets of data, not just to individual patients, and make use of a more sophisticated methodology. The researchers involved are typically not identical with the medical practitioners, whose actions contributed to produce the analyzed material. The potential bias inherent to individual perspectives is often sought to be counterbalanced *via* different kinds of triangulation (Keen and Packwood, 1995; Yin, 2009). Crowe et al. (2011) define the purpose of case study approaches as “to obtain an in-depth appreciation of an issue, event, or phenomenon of interest in its natural real-life context” (p. 1). Such case studies may thereby “provide insights into aspects of the clinical case and, in doing so, illustrate broader lessons that may be learnt” (p. 1). Crowe et al. (2011) further distinguish between three different types of epistemological approaches that may underlie case study research: The *critical approach* resembles the case studies in the medical tradition and has as its aim that the researchers involved openly question their own assumptions in the light of political and social factors such as power relations. *Interpretative approaches* aim at theory building and aspire to view the phenomenon in question from different perspectives in order to “understand individual and shared social meanings” (p. 4). The *positivist approach* usually focuses on “testing and refining” (p. 4) a pre-existing theory by studying variables established in advance and by contrasting them with the findings.

The approach taken in this article can be understood as standing at an intermediary position in between the above traditions. The same is true for the role of the author, who actively contributed to the collection and selection of the material, by which act he resembles the researcher in the medical tradition of case studies. In doing so, the author's position may be best described with Pols's concept of the “involved insider”, who engages in the practice of “contextual reflexivity” (Pols, 2006). However, there are also some features in which the study presented here overlaps with the social sciences' tradition of case studies: Accordingly, self-reflection on the part of the practitioners is a desideratum of what Crowe et al. (2011) categorize as “critical approaches” to case studies as well. Furthermore, the selection and discussion of the cases is not exclusively performed by the author himself here but supported by the constant change of perspective through the process of interactive interviewing. In addition, the selection of the cases and of an ED as the research site are based on theory, the findings are interpreted in the light of theory and are supposed to help its further development. This, in sum, constitutes a significant overlap with the positivist approach to case studies in Crowe et al.'s taxonomy.

### Case selection and analytical methods

For the case selection, the author, who is a psychiatric resident with 6 years working experience, engaged in “interactive interviewing” with three fellow residents from the same hospital (Tillmann-Healy and Kiesinger, 2001; Tillmann-Healy, 2003; Ellis, 2004; Adams, 2008). In interactive interviewing, participants mutually interview each other about their personal experiences with specific topics. The researchers, who engage in the process of interviewing, therefore, act as research participants themselves. The narratives which are thus produced are re-discussed and systematically reflected upon in the group. The aim of interactive interviewing is an in-depth understanding of another person's experience of complex and sometimes very personal matters. This can serve as a launching pad for a reflection which proceeds to more abstract concepts and starts the process of theory building or helps refining an existing one.

After a short introduction by the author into the concept of psychiatrization, all participants were instructed to think of “gray area-cases” they had personally encountered during shifts at the ED. In this respect, they were encouraged to focus on cases in which (a) fundamental questions arose about whether a displayed phenomenon truly fell within psychiatric expertise and/or whether (b) clinical decisions to handle a case in a medical way (e.g., by providing diagnosis and treatment) or not were outstandingly difficult and could have easily been decided the other way with equal plausibility.

From the collection of cases made in the interviewing process, two cases were selected. All participants agreed that they represented prototypical constellations for contacts in the ED in which practitioners experience fundamental doubts. These doubts were characterized as being much more about the question *if a psychiatric diagnosis was applicable at all* than about which diagnosis would fit best. In both cases, the practitioners' doubts arose mainly from a central question. In short, case (1) is an example of a patient who displayed some sort of psychopathology, but his symptoms seemed completely understandable and proportional when judged in the context of his biography and an ongoing marital crisis. Case (2) presented a situation which was highly dramatic at first sight and in which different understandings of suicidality and sadness were at stake.

Interpretation and analysis of the cases were performed in two steps. The aspects displayed immediately after the individual cases were mainly derived from the process of interactive interviewing. However, they have then been subjected to a more profound consideration by the author. The second step of interpretation consists of the analysis in the discussion part, which was exclusively performed by the author himself. It aims at summarizing generalizable features of the cases and connecting them to broader developments relevant to psychiatrization. Interpretation and analysis of the cases are, thus, both enabled by a theoretical framework and by the experience of the author as an involved insider, reflecting again the hybrid nature of this case study between the traditions of medical and social sciences.

### Cases

Within this section, two cases of psychiatric consultations in the ED of a medium-sized hospital with a psychiatric unit will be presented. All personal information about the help-seeking persons and their relatives has been anonymized. Details about specific persons or events were altered in a way that identification by third parties is impossible. Both cases originate from the hospital where the author and all participants of the interactive interviewing are currently or were working as psychiatric residents.

The hospital is located in the rural surroundings of Berlin/GER. The department of psychiatry comprises 94 beds for in-patients, among which 21 beds belong to the sub-specialty of psychosomatics. It includes three psychiatric day hospitals, three out-patient departments, and a home treatment-team and is part of the Brandenburg Medical School, a decentralized medical university established in 2014. The psychiatric unit is in charge of approximately 200.000 inhabitants of two counties, which belong to the federal state of Brandenburg. The ED is organized by nursing professionals and led by the specialty of internal medicine. Seven different specialties, including psychiatry, are involved in the acute treatment of a wide range of illnesses and injuries. Night- and weekend-shifts are typically covered by resident physicians, who are backed up by supervising senior physicians available on call.

The described hospital can be assumed to be neither especially prone to nor exceptionally resistant against psychiatrization and, thus, should most likely represent a (not yet quantifiable) average. For example, it is neither an ideological stronghold of biological psychiatry nor a place where standard psychiatric procedures are routinely undermined. Moreover, the selection of this hospital as the research site of this study enabled the participation of the author as an involved insider. From an ethical point of view, this also aligns with his conviction that research on psychiatrization from within psychiatry should include a high degree of self-critical thinking on the part of the practitioners.

#### Case 1: Depression or just a marital crisis?

Mr. A., a 51-year-old elementary school teacher, came to the ED with his wife, wishing to talk to a psychiatrist. He gave a very worried and somewhat burdened impression. In private conversation, he revealed that he sought help, because he was convinced to be suffering from a severe depressive episode. He stated that a self-test on the internet, belonging to an app for the online treatment of depression, told him so just that day. When asked about his complaints, Mr. A. described a depressive mood, a lack of energy, and a decrease in activity accompanied by loss of appetite, agitation, and sleeping problems. Mr. A. reported his complaints in accurate medical language, hinting at prior treatment experience and extensive engagement with the concept of depression. When asked about this, Mr. A. confirmed that his wife had received psychiatric treatment for depression some years ago. She also was the driving force prompting him to do a self-test for depression and behind his coming to the ED in the first place. He himself had no prior contact with psychiatrists or psychotherapists, except for the probatory use of an app and extensive search of information on the internet.

A more detailed examination of his complaints revealed that he had been suffering from an unstable mood over the last weeks, which did not appear to be consistently depressive. His primary concern was rather an inner restlessness, originating from intense worries about his future, which also impacted the quality of his sleep. His lack of appetite was only moderate, there was no sign of weight loss. Mr. A.'s level of energy was sufficient to keep doing his ordinary work and to take care of his 8-year-old daughter. He did not give the impression of being emotionally numb or unresponsive during the conversation and also confirmed that he had experienced some good moments during pleasurable activities with his daughter over the course of the last weeks.

All in all, standard psychopathological examination showed no signs of severe depression. Mr. A., relieved by this information, elaborated on his situation: 3 weeks ago, he had found out that one of his friends had been making advances on his wife. After some casual flirting *via* Whats-App, said friend openly confessed his love to Mrs. A. The latter was perplexed by this and showed the messages to her husband. She immediately replied to this declaration of love that she had no such feelings and requested the friend to stop contacting her. Nevertheless, Mr. A. remained deeply worried about this situation, because his last long-term relationship came to an end in a very similar way more than 10 years ago. In addition, his wife was 12 years younger than him. He had thus lived for years with the fear of losing her to a younger, more vital, and more exciting man. When asked about this, he admitted that he and his wife had encountered some conflicts before, because he tended to be suspicious and jealous when his wife met male friends or took part in leisure activities on her own.

While standard examination of psychopathology discouraged Mr. A.'s self-diagnosis, there were sufficient symptoms to justify the diagnosis of a mild to moderate depressive episode. However, the exploration of the context of Mr. A.'s complaints raised some doubts: From a strictly psychopathological point of view, the psychiatrist in charge remained unsure whether his symptoms, such as the described depressive mood or decrease in energy, appeared consistently or only sporadically, which would discourage a diagnosis of depression. In addition, she reported to have had a strong intuition that Mr. A.'s symptoms occurred as a very understandable, if not “normal” response to what had happened, and to how it had reopened emotional wounds. In the end, the psychiatrist who managed the case decided to diagnose a moderate depressive disorder according to ICD-10 (F32.1). Given that there was no sign of imminent danger and Mr. A. still seemed to handle many parts of his life quite well, she referred him to an out-patient service. Mr. A. also indicated that he would appreciate some pharmacological help for his restlessness and insomnia. The psychiatrist eventually handed out a small amount of Mirtazapine, an antidepressant with slight sedation as a welcome side-effect. She prescribed him a starter dose and suggested that his GP could augment it in 2–4 weeks. Although not being a formal standard, this proceeding corresponds to the clinical routines practiced by many residents, which are usually backed by their supervisors as medically rational.

Retrospectively, the psychiatrist in charge reported that Mr. A.'s case occurred to her as a typical gray area-case in which she could have refrained from psychiatric diagnosis and treatment with plausible reasons as well. She also stated that, thinking about it now, she would have preferred to take a second look at Mr. A.'s problematic 1 or 2 weeks later, before deciding about treatment and diagnosis. She did not consider this to be an option at the time, because she knew that keeping direct contact with the patient for watchful waiting would be impossible within the organizational structures of the ED and the hospital, where residents work in shifts and planned individual appointments are neither feasible nor reimbursable. In the following process of interactive interviewing, several other factors increasing the likelihood of a decision in favor of a psychiatric management of this case became visible: Mr. A. had a very clear notion of the nature of his complaints. He reported them in psychiatric vernacular and cited a self-test as proof of credibility. Furthermore, his wife had reassured him that he might be in a similar condition she used to be in when she was labeled depressive. All these factors, on the one hand, not only pre-formed Mr. A.'s own assumptions about his condition, but also shaped how he experienced and displayed his concerns as symptoms of depression. On the other hand, his expression and articulation of his complaints were very likely to influence the perception of the psychiatrist in charge, as clinicians may be susceptible to buzzwords and are trained to be on the watch for signs of hidden depression against the background of its widely postulated under-recognition and the dangers lying therein (e.g., Demyttenaere et al., 2004; Kohn et al., 2004; Merikangas et al., 2011; Werlen et al., 2020).

In sum, Mr. A. and his wife presented with the more or less explicit wish for a medical diagnosis of Mr. A.'s condition followed by medical treatment. Their expectations and desires were thus expressed in a way, that was inviting for psychiatrization. By coming to the ED, the couple also underlined that they were looking for *immediate* help and judged their problem to be urgent, at least too urgent to risk the typically long waiting times for an appointment with an out-patient psychiatrist or psychotherapist instead. Although the psychiatrist in charge reported that she did not feel directly pressured, she confirmed that she sensed that not giving in to her patient's expectation, refusing or postponing pharmacological treatment, would have entailed a time-consuming discussion and probably even generated an open conflict. In the end, clinical diagnosis and the start of treatment largely confirmed Mr. A.'s preexisting assumptions and may very likely have cemented his perception that his complaints originated from or were part of a mental disorder.

During the process of interactive interviewing, the participants tried to figure out what a less psychiatrizing intervention could have been like: One option for the psychiatrist in charge might have been to communicate to Mr. A. that she found his current health concerns and worries adequate, especially in the light of his prior experience with existential crisis in a similar situation. She could also have emphasized the harmlessness and presumably transient character of this episode of crisis. Furthermore, she could have offered a non-pathological explanation by suggesting to understand the problematic primarily as a marital crisis. This would have entailed a shift of perspective to the couple, instead of singling out Mr. A. as the “ill individual”. Against the backdrop of a systemic instead of an individualistic concept, the psychiatrist on duty could have also encouraged Mr. A. to make his feelings and fears transparent to his wife (Fryszer and Schwing, 2014). If enough time had been available, she even could have started this process by inviting Mrs. A. to a short conversation while still in the ED. Thus, she could have emphasized the importance of open communication and of spending time with each other to the couple's relationship.

Other questions that surfaced during interactive interviewing were in which respect the intervention of the psychiatrist on duty was helpful and what other course of action could have been beneficial to Mr. A.'s situation. As there was no follow-up of his case, these questions remained purely hypothetical. Of course, the antidepressant may have had an immediate soothing effect and later-on possibly lifted Mr. A.'s mood. But opposed to the psychiatrization, to which Mr. A. has been submitted by means of the diagnosis and the subsequent treatment, enabling open communication about the hidden, maybe unconscious motives behind the couple's desires for psychiatric help could have been beneficial to both of them—especially in the long run. Moreover, from Mr. A.'s point of view, the diagnosis of a severe mental disorder could have been understood as symbolizing the severity of his suffering and, as such, served as proof of affection for his wife. Mrs. A., by contrast, by urging him to seek psychiatric help, signaled to her husband that she cared for him and that she took his suffering seriously. Seeking to make these complex dimensions of the situation visible to the couple might have been more helpful than diagnosis and pharmacological treatment. The interview group agreed that shifting the focus of attention to pharmacological treatment (e.g., by inducing intensive thinking about effects, side-effects, dosage, ability to drive, becoming addicted, etc.) could even have been counterproductive by distracting from what really was at stake. The processes of understanding and reconciliation which seemed to be central to Mr. and Mrs. A's crisis could thus have been hampered.

#### Case 2: Suicidal or just sad?

On a Sunday afternoon, Ms. B., a barely 19-year-old apprentice in web design, was brought by ambulance to the ED from nearby Berlin. The paramedics announced her to be suicidal but cooperative. The ambulance was accompanied by the police, who had forced their entry into Ms. B.'s apartment, after having been warned that she might be in the immediate danger of suicide.

Ms. B. agreed to talk with the psychiatrist on duty. She was genuinely polite and gave a sad and somehow intimidated impression. In private conversation, she explained that everything went terribly wrong that day, actually not only that day but over the course of a longer period of time prior to her admission. According to her, it had all started with the sudden death of her father 4 months before. Her father had been suffering for several years from a carcinoma with a relatively good prognosis. It used to be under control, but, all of a sudden, severe complications occurred, and he died within a few days. At about the same time, Ms. B. moved from the suburbs to more central Berlin. This change of residence was planned in advance due to her apprenticeship in Berlin. This move initially appeared to help her to cope with her father's death by providing her some inner distance and symbolizing a new, positive step in her life. Furthermore, her relationship with her boyfriend used to help her through this difficult time. They had had a long-distance relationship, but had managed to see each other every other weekend for more than a year.

Ten days before her admission, her boyfriend told her that he had fallen in love with her best friend and therefore wanted to end their relationship. For Ms. B., this came totally unexpected and also struck her as quite absurd, because she knew that the friend in question was not very fond of her boyfriend. Thus, Ms. B. felt heart-broken, alone, and terribly sad. Because her boyfriend lived in a distant city, she could not even talk to him in person. She sent him messages and sometimes called him for a couple of minutes in a desperate attempt to understand what had gone wrong and how she could fix it. For the 4 days prior to her admission, she had shut down all contact with her ex, because she felt that it was dragging her down. She had realized that she was unable to change his decision. However, earlier that day he had called her to ask if she was alright. Ms. B. was emotionally overwhelmed by this and told him that she was feeling terrible, and that she did not know how to go on with her life. She then hung up, shut down her phone, laid down on her bed, and turned on loud music on her headphones. One hour later, the police came crashing through her door. It turned out that, when her ex had realized that he was unable to reach her on her mobile phone, he had called her mother, who decided to alert the police out of deep worry for the life of her daughter.

In close examination, Ms. B. confirmed intense feelings of sadness. She did not sleep well, had troubles concentrating, had little appetite, and was not enjoying her hobbies very much over the course of the last 10 days. However, she reported that she had managed to keep doing all the tasks related to her apprenticeship. Moreover, spending time with friends had done her good. Sometimes, there were even moments when she started to sense that she would eventually overcome her broken heart and soon be fine again. She admitted that she had been thinking a lot about suicide over the last days. Such thoughts were entirely new to her. She experienced them as both frightening and somehow soothing to her inner pain. Nevertheless, she argued quite convincingly that it was very unlikely that she would actually commit suicide: She had never engaged in precise planning or preparation, she was rather the type for overthinking than for impulsive action and she could never do such harm to her mother and her younger brother, who had just suffered the tragic loss of her father.

The psychiatrist on duty asked Ms. B. how she would like to proceed. Ms. B. told him that she just wanted to go home after this nightmarish trip to the hospital and maybe do some sports or read a book. The psychiatrist suggested that her mother could pick her up, but she asked for not involving her mother any further. They agreed that there was no need for medication at the moment and that Ms. B. should consider getting some support through a psychotherapy at some point, should she feel unable to cope alone with her situation in the future. The psychiatrist in charge showed her how to look for therapists on the internet. After short communication with the supervising senior doctor, he released Ms. B. from the ED under the condition of a telephone call the next day to confirm that she would be alright.

The psychiatrist on duty reported that he had very ambivalent feelings about the case of Ms. B. and about how to manage it. On the one hand, when strictly following the ICD-10 manual, neither the time criteria nor the symptom criteria for a depressive episode were fully satisfied (World Health Organization, 1993). In addition, there was no sign of any other preexisting mental disorder. On the other hand, it was obvious that Ms. B. was not well for very good reasons. Giving her no diagnosis at all would have felt to him like failing to acknowledge this fact. Eventually, the psychiatrist in charge decided to label her problematic as an adjustment disorder (F43.2), which is one of the very few diagnoses in the ICD characterized by being self-limited, directly caused by stressful life events, and free from strong neurobiological assumptions (Bachem and Casey, 2018; O'Donnell et al., 2019; Strain, 2019). Furthermore, it was clear that a diagnosis was required for the financial compensation of the hospital's services.

The interview group agreed that the case of Ms. B. was a gray area-case in which many colleagues, and the participants themselves as well, could have decided quite differently with very convincing arguments. The most difficult decision in this case was identified as being not which diagnosis would be accurate, but whether to admit Ms. B. to the psychiatric ward, or to discharge her from the ED. In this specific case, the psychiatrist on duty consulted his supervisor, because he was aware that discharging a patient who had been announced as suicidal and brought in by the police was rather unusual compared to the clinical routines. Finally, they worked out together that an immediate discharge was clinically justified and that, in the absence of legal criteria for an involuntary admission, the wish of the patient had to be paramount.

Several reasons why professionals could be inclined to favor the hospitalization of Ms. B. surfaced in the group discussion, among which risk reduction was the most salient: Although it may have been small, the risk that Ms. B. would eventually commit suicide—maybe in an impulsive act after another destabilizing call by her ex—could not be ruled out entirely. In-patient treatment might have diminished this risk for Ms. B. as well as the legal risks for the psychiatrist on duty. In this specific case, he and his supervisor consciously accepted to take a (legal) risk by letting Ms. B. leave the hospital.

The interview group agreed that they themselves often felt a strong intuition that a person suffering to the point that she experiences suicidal thoughts *must have some kind of depression* regardless of the diagnostic criteria. This intuition may be rooted in psychiatric commonplace knowledge, e.g., that the majority of suicides is related to mental disorders and that depression, especially, constitutes a risk factor (Bertolote et al., 2004; Ferrari et al., 2014; Bachmann, 2018). But this intuition also seems to correlate with a widespread cultural assumption that echoes still existing taboos about suicide or, to be more abstract, about death and mortality in modern westernized societies in general (Becker, 1973; Ariès, 1974; Améry, 1976; Elias, 1985; Jacobsen, 2016). Against the backdrop of psychiatrization, the assumption that suicidal thoughts imply mental disorders could be problematic, as it seems to suggest the categorical exclusion of suicidality from the realm of what is “normal”. Instead, suicidality is thus conferred to the realm of health problems and relegated to psychiatry as the medical discipline in charge of handling it. However, the taboo on suicidality could also be understood as a hint at a high, but hidden prevalence of suicidal thoughts and behaviors, possibly being more “normal” features of human life than society and institutional psychiatry believe or wish them to be.

From a broader perspective, the case of Ms. B. may point to even more cultural issues. As Horwitz and Wakefield (2007) claim in their seminal work “The Loss of Sadness”, there is a deep running cultural deficit to perceive sadness through a non-medicalized gaze. This deficit originates from a cultural vacuum of concepts which would allow to perceive intense sadness, grief, and human suffering as something different than a mental disorder and also becomes palpable in Ms. B.'s case. Following Horwitz and Wakefield, it has become nearly unthinkable that suffering from a sadness deep enough to consider suicide could be anything other than the manifestation of a depression. Thus, soft factors, such as the cultural repository of concepts of sadness, might pave the way for diagnosis and treatment, even in cases where the diagnostic criteria of depression are not entirely satisfied.

To conclude the discussion, the interview group deliberated on the hypothetical question whether admitting Ms. B. to the psychiatric ward would have been an act of psychiatrization or not. The participants referred to their experience that hospitalization usually goes along with giving patients a rather severe diagnosis (such as depression compared to adjustment disorder). In addition, inpatient treatment is very prone to include medication. Both of these aspects could have a strong psychiatrizing effect. They could constitute the starting point of a prolonged and recurrent use of hospitals and other psychiatric services. This could have entailed a gradual redefinition of Ms. B.'s concept of herself and her problems in psychiatric terms. In the case of Ms. B., prolonged hospital stays were perceived as a realistic risk, since she was in an intrinsically difficult period of her life with challenges such as her move out from home and the start of a new professional career. Moreover, she had to cope with the premature death of a parent and the emotional turbulence of lost first love. In her case, the interview group agreed that in-patient-treatment could have had the effect of rather distracting her from tackling these challenges. By contrast, discharging Ms. B. from the ED with a vague recommendation of seeing a psychotherapist was perceived as a rather supportive move which could potentially help her to process her grief and to re-calibrate her life. Furthermore, the relatively pale and obscure diagnosis of adjustment disorder was judged to be less of an entry point for a psychiatric re-shaping of Ms. B.'s identity than a depressive episode, which was seen as inviting much more for identification and becoming a lived reality.

## Discussion

Studies with a double focus on the ED and on psychiatry as an institution are rare. Literature from the psychiatric discipline is mostly concerned with the practical management of cases perceived as psychiatric emergencies (e.g., Chanmugam et al., 2013; Nicholls, 2015; Riba et al., 2016). In addition, there is a rich literature dedicated to the broader topics of violence reduction in psychiatric settings (e.g., Gerdtz et al., 2020; Biondi et al., 2021) or on involuntary hospitalizations (Weich et al., 2017; Sheridan Rains et al., 2019; Walker et al., 2019), which only touches upon the ED in some respect. In the social sciences, there is a long tradition of inquiry into cases of involuntary commitment to psychiatry and their underlying social determinants. For instance, being black, male, or arriving with the police has been found to increase the risk of involuntary hospitalization (Jeffery, 1979; Horwitz, 1982; Rosenfield, 1982, 1984; Lindsey and Paul, 1989; Way et al., 1993). However, more recent studies, such as Lincoln (2006), indicate the need for a paradigm shift: Conflicts in and around the ED may be increasingly about patients' interests in getting access to psychiatric care and the professional denial thereof. In times of growing economic constraints on hospitals, people from vulnerable groups might, thus, be much more likely to be exposed to the risk of being excluded from adequate care than of being socially controlled by involuntary hospitalization. In a similar vein, Lane (2020) points out how psychiatric diagnosis, as the key to medical care, has become an increasingly contested terrain with intense negotiations taking place between professionals and help-seekers in all settings which involve psychiatric assessment. Although psychiatric diagnosis is traditionally thought of as “stigma-laden” (Thornicroft et al., 2009; Henderson et al., 2014) and thus seems intrinsically highly undesirable, these negotiations point to the fact that it may also be appealing for people to receive a psychiatric diagnosis under some circumstances. Motives for the desire for a psychiatric diagnosis may be diverse but could partly be illuminated with Parson's classic concept of the sick role, that shifts the main responsibility to solve the then medically framed problems to the healthcare system and deflects moral judgment and guilt from the individual (Parsons, 1951). This may be especially attractive in cases such as those of Mr. A., when socially unwanted behavior would otherwise be explained as personal weakness or flaws in character (Moncrieff, 2020).

### Psychiatrization in the ED

Two individual cases from the ED of a middle-sized hospital with a psychiatric unit have been presented and interpreted above. These cases have in common that it was deeply uncertain to which degree the presented phenomena fell within psychiatric expertise or not. Although gray area cases of this type may be frequent, many clinical psychiatrists would most certainly insist that the vast majority of patients presenting in the ED are either unambiguously “non-psychiatric cases”, for example when an underlying somatic pathology can be identified or when symptoms clearly do not reach the threshold for diagnosis, or “psychiatric cases” in the sense that diagnostic criteria for a mental disorder are obviously fulfilled. Also, many people coming to the ED have long histories of psychiatric treatment under a certain diagnosis, during which their problematic has been assessed and reviewed by several experts. In these cases, the medical act of conferring the right diagnosis might be of minor, rather abstract importance in the ED from the practitioner's point of view, compared to much more concrete matters such as finding the right setting for acute treatment or improving insufficient individual medication. The gray area cases analyzed within this article thus only represent a certain part of the every-day work of psychiatrists in the ED, but, as will be demonstrated, a part that bears special relevance for advancing the understanding of psychiatrization in mental health care.

The overall results of this case study corroborate the recent paradigm shift in research toward emphasizing the agency of help-seekers and their relatives in the ED. They align well with Lincoln's (2006) and Lane's (2020) observation that psychiatric diagnosis and treatment in the ED may often be the result of complex negotiations, but also add to their findings by providing detailed insights into the negotiation process and the situational and ex-post reflections of psychiatric professionals. However, the central research question of whether the ED as an area of contact between psychiatry and society could be a place where psychiatrization may emerge in an observable way remains difficult to answer.

In the case of Mr. A., it can be argued that psychiatrization appeared in the specific sense that a certain problem, whose nature was fundamentally unclear, was claimed to fall within psychiatric expertise by its interpretation, diagnosis, and treatment as depression. In other words, through diagnosis and treatment Mr. A.'s depression *came into being* in an (at least) three-fold sense: (a) as a subjective conviction, which may gradually become a lived reality, (b) as an intersubjectively shared social reality (e.g., when relatives or professionals refer to the diagnosis and perceive a person through a certain diagnostic category), and (c) as a legal entity, which entitles to health care or other means of support. This constellation is comparable to the case of Ms. B., although she did not receive specific treatment and it is unclear whether the unspecific and rather pale diagnosis of an adjustment disorder really has the potential to become a subjective conviction that could evolve into a lived reality. Nonetheless, the doubts about the re-shaping of personal identities through psychiatric diagnosis and treatment in her case point to an important aspect: When it comes to individual cases, psychiatrization and its effects may be much better observable in a longitudinal perspective than by research designs which only cover a very limited timeframe. Without a follow-up on the cases, there is no way to know if Mr. A. ended up rejecting his medication and diagnostic label, or if Ms. B. went to an out-patient-psychiatrist and requested and received antidepressants. However, the above cases, as well as case study approaches to psychiatrization in general, may be useful to show situations which are crucial to individuals, since they constitute their first point of contact with the mental healthcare system in a situation of crisis. In this context, the explanations offered by practitioners and their decisions may significantly increase or decrease the chances of inducing profound changes within the identities of help-seekers and kick-start psychiatric patient-careers by means of—but not limited to—diagnosis, service-use, and medication.

### Drivers of psychiatrization

**Figure 2:**
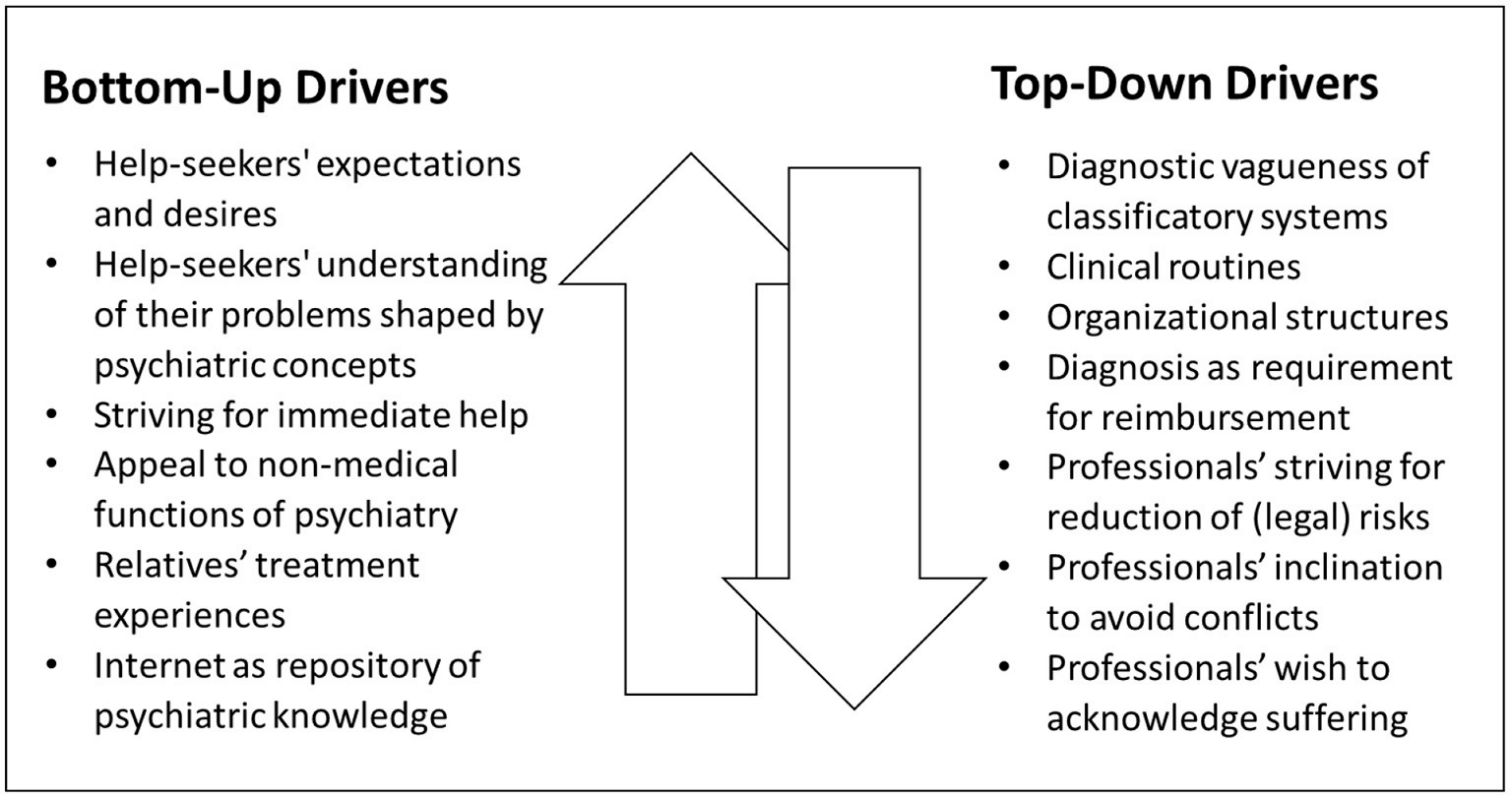
Drivers of bottom-up and top-down psychiatrization in the ED. **Bottom-up drivers**: (a) Help-seekers' expectations, encompassing their own diagnostic assumptions, and more or less specific desires for psychiatric diagnosis and treatment. (b) Help-seekers' understanding of their own problems that may have been shaped by psychiatric concepts and delineated by means of psychiatric vernacular. (c) Help-seekers' striving for immediate help, that may create an atmosphere of urgency even when watchful waiting would be suitable. (d) Help-seekers' appeal to psychiatry for non-medical functions, which may be related to its implicit (pedagogical, symbolic, ritualistic, mediating, etc.) dimensions. (e) Treatment experiences of help-seekers' relatives who may act as multiplicators of psychiatric expertise by providing psychiatric interpretations and giving recommendations based on how they were previously treated and what they were told by psychiatric professionals. (f) The internet as a repository of psychiatric knowledge, which is easily accessible and often consists of strongly simplified, popularized versions of expert-knowledge. **Top-down drivers**: (a) The diagnostic vagueness of psychiatric classificatory systems, that encourages ascribing diagnoses when operating in the gray area and opens up a space for negotiation between professionals and help-seekers. (b) Clinical routines that favor medication or hospitalization, e.g., when alternatives are not available in the ED-setting and finding individual pathways for psycho-social help is more time-consuming than following standardized medical procedures. (c) Organizational structures that impede watchful waiting and, thus, encourage diagnosis and the immediate initiation of (pharmacological) treatment, e.g., when psychiatrists working in the ED have no means to make follow-up appointments or cannot be sure if help-seekers will be able to see an out-patient psychiatrist soon. (d) Diagnosis as requirement for the reimbursement of services, putting economic pressures on hospitals and EDs, which increases the likelihood that people seeking help in situations of distress will receive a psychiatric diagnosis. (e) Professionals striving for risk reduction, including (their own) legal risks when underestimating or missing potential dangers, which may considerably lower the threshold for hospitalizations, diagnosis and treatment. (f) Professionals' inclination to avoid conflicts, which are likely to arise when help-seekers' (or their relatives') expectations and desires for a certain diagnosis or treatment are not met. (g) Professionals' wish to acknowledge and dignify human suffering through diagnosis and treatment, e.g., when watchful waiting would cause disappointment and feel like disregarding the problem causal for coming to the ED.

Despite lacking a long-term perspective, the above cases contribute to deepening the understanding of psychiatrization by giving insights into the considerations of psychiatric practitioners, by outlining their range of action and revealing some of the factors that influence their decisions. Several factors that arguably increase the likelihood of psychiatrization in bottom-up or top-down ways became visible, of which not all have directly impacted the two above cases. However, these factors were part of the practitioners' considerations either in the original situation or in retrospective. Many of them might be generalizable in the sense that they may favor decisions with a higher risk of psychiatrization compared to a less psychiatrization-prone approach also in other cases and in different settings. In the terminology of the comprehensive model (see Figure 1) they, thus, can be classified as *drivers of psychiatrization* that either predominately run top-down or vice versa (see Figure 2).

In addition to the drivers listed above, several soft factors, which shape the context for both top-level and bottom-level agents, surfaced in the cases. These contextual factors are difficult to categorize. They may encompass general notions of normalcy that circulate in society as well as concepts which are culturally available to explain, understand, and give meaning to human suffering. Furthermore, there are many smaller or larger narratives which provide interpretations to crisis-like situations and may also determine what seems the right thing to do to when in such a situation. It is possible that these notions, concepts, and narratives are heavily influenced by psychiatric expertise, e.g., when crystallized into the form of a classificatory system. However, it seems convincing to assume with Hacking that there are strong reciprocal connections between society and psychiatric knowledge (Hacking, 1985, 1995b). Following this train of thought, even the act of creating a psychiatric classificatory system would be strongly impacted by assumptions about human suffering and about what is to be considered “normal” or “pathological” that were already present in society and nurtured by many other sources apart from psychiatry such as religion, spirituality, art, and the media.

In addition, a few other drivers appeared for which it seems unclear whether they primarily work in a bottom-up or a top-down way. For example, it is difficult to categorize the role of paramedics, who have a general medical training but are not specialized in matters of mental health. They, thus, do not clearly belong to the group of experts on the top-level. However, when called to an emergency, they might happen to be the first professionals who offer an interpretation of a situation or (health) problematic. This preliminary label may influence how a case is perceived and managed, e.g., whether patients are intentionally brought to a psychiatric unit, whether their chief complaint is announced as being psychiatric to the triage nurses, or whether help-seekers are directly handed over to psychiatrists. In a similar vein, many patients are brought to the ED by the police without the involvement of any medical professionals. Although police officers have no special medical training, which would support categorizing them as laypeople, they represent the state's authority and have the power to instigate an involuntary commitment to psychiatry, which would justify classifying them as agents of top-level-psychiatrization.

Many of the various drivers of psychiatrization which surfaced in the above cases would deserve a more detailed reflection. Given the scope of this paper, only four of them were selected for further considerations. This selection comprises (a) classificatory systems and diagnostic vagueness, (b) multiplicators of psychiatric knowledge, (c) non-medical functions of psychiatry, and (d) the power of narratives. While (a) mainly represents a top-down driver of psychiatrization and (d) is rather a contextual factor, (b) and (c) were selected to underline the significance of bottom-up psychiatrization. All four drivers are important in the context to the ED but may also be generalizable in the sense that they are likely to play an important role in many more cases than the above and in different settings. Thus, they could broaden the understanding of how and why psychiatrization may take place wherever mental health professionals need to determine whether the persons seeking help should receive a psychiatric diagnosis and treatment or not.

#### a) Classificatory systems and diagnostic vagueness

As has been exemplified above, diagnosis seems crucial to either inducing a psychiatric interpretation, management, and treatment of a specific problem, or not. In spite of this paramount importance of diagnosis as a kind of watershed moment, the available diagnostic manuals (ICD and DSM) have, since their introduction, been ongoingly challenged for their poor reliability and questionable validity (Frances, 2013; Regier et al., 2013; Lilienfeld, 2014; Wakefield, 2015; Fried, 2017; Fried et al., 2020).^1^ This weak spot may be especially problematic when it comes to the increasing number of patients utilizing mental health services for what professionals perceive as mild or moderate disturbances (Hart, 1971; Wang et al., 2007; Olfson et al., 2019). With the mere presence of clusters of symptoms as defining criteria for diagnoses, and with little regard given to symptom severity (notwithstanding poor means to objectify severity), classificatory problems when using ICD or DSM may emerge, in particular, if some symptoms are to be found but they do not seem severe.

For instance, the diagnosis of a depressive disorder was plausibly applied to case 1 and could have been plausibly applied to case 2 as well. The definition of a depressive episode includes a broad spectrum of relatively unspecific symptoms of mental distress. Against this background, it appears only logical that anyone facing a larger life problem or any kind of personal loss will display at least some of these symptoms to a certain extent (Horwitz and Wakefield, 2006; Horwitz, 2015; Wakefield, 2015). Since this certain extent is not quantified in DSM or ICD, psychiatrists may tend to rely on vague overall impressions in order to fathom whether a certain psychological reaction is proportional or whether it is to be considered as excessive. However, when operating in such a space of diagnostic vagueness psychiatrists' decisions may very likely be influenced by factors that promote psychiatrization such as the above listed drivers. Of those, the role of the economic necessities of hospitals should not be underestimated. Since mental health services in many countries depend on psychiatric diagnosis for their reimbursement, this constitutes a strong systemic incentive to apply psychiatric labels to people seeking help in the ED as well (Batstra and Frances, 2012b,c). In many cases, this labeling may only be possible, when classificatory systems comprise unspecific diagnostic codes and criteria for diagnosis that can be handled quite loosely. The funding of clinical psychiatric settings may, thus, rely to a certain extent on the vagueness of classificatory systems.

The diagnostic vagueness of the existing classificatory systems, which may become most salient at their margins, might, thus, be fundamental to the psychiatrization of persons in distress in the ED and in other settings. Consequently, the creation of diagnostic manuals that generate diagnostic vagueness and encourage psychiatric diagnosis, when operating in a gray area, may constitute a powerful driver of top-down psychiatrization.^2^ However, and somewhat paradoxically, the vagueness of the classificatory systems may also enable bottom-up psychiatrization, since it opens up a space to negotiations between professionals and help-seekers. Nonetheless, the manual's construction, interpretation, and application ultimately lie in the hands of psychiatrists. Therefore, it seems legitimate to classify diagnostic vagueness mainly as a top-down-driver of psychiatrization.

Nevertheless, this criticism risks being misleading. The fact that a person's symptoms fit into the frame of a certain diagnostic category does by no means prove that an individual is “mentally ill” in the sense that there is a distinct disease entity from which the individual is suffering. The ontological foundations of psychiatry are far from being unequivocal. In other words: The formally correct application of a diagnostic category on an individual case does neither prove that the applied specific category is valid nor that the overall assumption that mental disorders exist and can be classified is true. However, the complex scientific and philosophical debate about the existence, or reality of mental disorders, which has accompanied modern psychiatry from its beginnings (e.g., Szasz, 1974; Bolton, 2008; Hyman, 2010; Graham, 2013; Kendler, 2016) cannot be settled satisfactorily within the confines of this article. For the purpose of this analysis, it seems sufficient to acknowledge that, once a psychiatric diagnosis is ascribed to an individual, a mental disorder becomes real in the aforementioned three-fold sense of a subjective, lived reality, an intersubjectively shared social reality, and a legal entity.

Following this line of argument, the main criticism to be leveled against classificatory systems is not that they are weak tools to tell the people who “really” are mentally ill in an ontological sense from the people who are not.^3^ Once a diagnosis is made, a person who does not actively refuse it *is really mentally ill* in the above three-fold sense. Moreover, the point is that classificatory systems put up very low barriers to bring many people with mild, unclear, and unusual (health) complaints into being as mentally ill subjects. However, from a clinician's perspective, this criticism may contain some ambivalence: As also the most severe and disabling conditions might begin with mild or unclear symptoms, early detection of those with a high risk of developing severe and enduring mental disorders can potentially also be an opportunity to intervene before mental distress erupts into a full-blown crisis or becomes chronic (Trivedi et al., 2014; Arango et al., 2018). A more rigorous, less vague classificatory system with higher thresholds for diagnosis thus could also mean to curtail the chances for early intervention or even prevention of severe mental distress.

#### b) Multiplicators of psychiatric knowledge

As psychiatrization implies the increasing influence of psychiatric concepts in society, it may be worth asking what the above cases contribute toward an understanding of how psychiatric knowledge circulates between professionals and lay-people. The case of Mr. A. hints at two different ways how psychiatric knowledge may become a determining factor of how people cope with a crisis-like situation even without consulting a psychiatrist.

First, it is noteworthy that the admission of Mr. A. to the ED was prompted by his own internet research, through which he came across an online intervention for depression. As in many other aspects of life, the internet has become an important repository of easily accessible knowledge. This also holds true, when it comes to the matter of mental health (Christensen and Griffiths, 2000; Baker et al., 2003; Hesse et al., 2005; Loos, 2013). However, unless such research is very specific, the information suggested, when one types in general terms like “depression” and “treatment”, are very likely to represent the hegemonic biomedical positions, as the algorithms of search engines obey to the laws of the attention economy (van Dijck, 2010; Morozov, 2012; Bozdag and van den Hoven, 2015). These may include exaggerated guesses about the prevalence of mental disorders as well as an increasing number of online interventions that encourage self-diagnosis and are aggressively marketed by private companies (Beeker and Thoma, 2019; Cosgrove et al., 2020).

It is easy to imagine that stumbling upon such information or “help” in a very early state of making sense of one's own “not feeling well” can impact subjective interpretations and expectations. What is striking about Mr. A. is that he described his condition in accurate psychopathological terminology and that he arrived at the ED with the clear expectation that his self-diagnosis would be confirmed. Prior to any contact with a psychiatrist, he already started to categorize, perceive, and experience his ailments as a matter of mental health or, more specifically, as symptoms of depression. Although it remained empirically unclear how much his internet research contributed to this psychiatric re-shaping of his identity, as compared to other influences, his case gives a rough impression of how the internet could be an important multiplicator of psychiatrization by popularizing psychiatric concepts, encouraging identification with them, preforming expectations about diagnosis and treatment, and disseminating the vernacular of psychiatry in society.

Second, it is also remarkable that Mr. A.'s notion of being mentally ill was firmly supported by his wife. She had prior treatment experience and was fairly convinced that her husband was in need of psychiatric treatment as well. The role of Mrs. A. points to an important aspect as to how psychiatric knowledge circulates in society. Laypeople who have experienced psychiatric treatment themselves and who have, as opposed to psychiatric “survivors” (LeFrançois et al., 2013), accepted their psychiatric labels, might act as *spreaders of psychiatric concepts*. They may impact their relatives, friends, colleagues, etc. by providing psychiatric interpretations to problems or health concerns and by promulgating recommendations based on their own experiences as well as on what they were told by professionals. They, thus, may contribute to disseminate psychiatric knowledge (or: a personalized, possibly simplified version of psychiatric expert knowledge) and ideas of how to help someone in mental distress. In doing so, ways of how to react to individuals' crises may be inscribed into the body of common-sense knowledge of a society. Laypeople with treatment-experience, thus, may act as multiplicators and reinforcers of bottom-up psychiatrization by iterating and spreading top-down expert knowledge (Beeker et al., 2020). They thereby participate, presumably for benevolent reasons, in ingraining patterns of perception, interpretation, and action, which are essentially shaped by psychiatry, into their social networks and society as a whole.

#### c) Non-medical functions of psychiatry

Many admissions to psychiatry may be prompted by relatives of the individuals in distress. Their motives for this can be diverse: As in both above cases, the primary reason is frequently the honest belief in the necessity of psychiatric treatment and the assumption that it would be helpful. But relatives may also wish to share responsibility with professionals, as in case 1, or even to shift the main responsibility to institutional psychiatry.

Apart from this, an admission to psychiatry may sometimes also be resorted to for *pedagogical* reasons. In case 1, bringing Mr. A. to the ED also signifies that his level of worrying exceeded what Mrs. A. perceived as tolerable. The case of Ms. B. provides a much stronger example. Her encounter with psychiatry was initiated by her mother and her ex-boyfriend as a reaction to her implicit threatening with suicide. Her admission to psychiatry, including the dramatic act of the police forcing their entry through her door, can, thus, also be understood as a very powerful statement that it means crossing a red line to utter suicidal thoughts and then shut off one's phone. Beside more benevolent motives, psychiatry was used here to teach Ms. B. a pedagogical lesson, namely that she went too far in a way considered not “normal” and intolerable.

In contrast to this pedagogical function, seeking help from a psychiatrist can also have a more positive *symbolic* or *ritualistic* dimension. When Mr. and Mrs. A. decided to come to the ED, their decision was based on the mutual acknowledgment of a need for change. For them, seeking professional help was not only intended as a first practical step, *it was* an act of reconciliation in itself. In this context, psychiatry is appealed to as an abstract authority which bears witness to the agreement that “something has to change” and to acknowledge the sincerity of this desire for change. In some cases, the role of the psychiatrist may, thus, rather resemble the role of a priest, or of a notary, than the role of a physician. Moreover, psychiatrists may often also act as *mediators*, not only witnessing, but actively facilitating the reconciliation of people coming to the ED.

In sum, psychiatry may be appealed to for non-medical functions as well, e.g., as a pedagogical, ritualistic, or mediating authority. These functions of psychiatry seem to be rather implicit reasons for consultations. When compared to the often much more explicit display of symptoms, it can be difficult for psychiatrists to discover whether or not non-medical motives predominate in a specific case. From a more general perspective, it is debatable whether psychiatry is (or: should be) equipped to handle such needs or if these kinds of needs are misdirected and should be delegated to other (therapeutic) professionals. However, in relation to psychiatrization, desires for genuinely non-medical services may become problematic, at least when they are answered by diagnostic and therapeutic reflexes.^4^ Such a reflex response may be considerably facilitated by the above criticized vagueness of the classificatory systems. Laypersons' somewhat misguided desires and psychiatrists' professional tendency to perceive, classify, and handle them in a medical way may, thus, also be contributing to increase the risk for psychiatrization in emergency care settings.

#### d) The power of narratives

Narratives appear to belong to the “soft” factors which may pave the way for a psychiatric interpretation of distress and crisis. A broad corpus of scholarship from the humanities and social sciences has stressed the importance of narratives as meta-structures through which people make sense of themselves, other people, or different aspects of life (Todorov, 1969; Gubrium and Holstein, 2009; Frank, 2010; Puckett, 2016; De Fina and Georgakopoulou, 2019). In this sense, narratives are a universal feature of our social world and a constitutive part of each individual's identity. Accordingly, it is obvious that storytelling is also omnipresent in psychiatry. This starts with listening to the (life-)stories of patients, which are then condensed and retold, when the cases are presented to colleagues or written down in an anamnesis or epicrisis. When listening to, telling, retelling, and writing down stories, aspects or facts are brought into a comprehensible order, following unconscious, but influential rules of how to construct a narrative. In the end, people tend to produce logical and coherent stories, which are implicitly also tailored to their aesthetic and dramaturgic inclinations. Moreover, such stories tend to have similar climaxes, or punch lines as the dominant narratives circulating in society (Gubrium and Holstein, 2009; Frank, 2010; Puckett, 2016).

From a narrative perspective, both cases have all the ingredients of a very compelling story: Mr. A used to lead a happy life with his beloved wife and his daughter, when he suddenly realized that he was on the verge of losing his wife to a younger man. But he was wrong: With the help of the internet, his caring wife, and a competent psychiatrist he found out that he was just in a state of depression, which was casting a shadow on his mind and soul. In Ms. B's story, the protagonist used to be a thriving young woman who was looking forward to moving to the big city and standing on her own feet, when she tragically lost two of the most important figures in her life, one after the other. In reaction, a deep depression entered her life and she became suicidal, but overcame this crisis through the help of psychiatry.

In both constellations, depression figures as an easy-to-understand cause for the protagonists' encounter with psychiatry. But it also is the meta-structure that gives meaning to everything before and after their encounter with psychiatry and that makes the overall plot convincing. Moreover, in both cases, “depression” nearly materializes into an independent agent which intrudes into a happy state (“disruption”) and has to be expelled before again reaching the former equilibrium, a structure that vastly resembles Todorov (1969) influential theory of narratives.

What may be most important when analyzing the above cases in the light of narrative theory, is that the strong intuition that Mr. A. and Ms. B. must have some kind of depression may originate less from clinical evidence but rather from the human inclination to tell compelling stories. Such stories connect well with the culturally available narratives which serve as their prototypes. Clinical psychiatrists, thus, may sometimes be at risk of succumbing to the charm of compelling narratives which may only seem to be based in psychiatric nosology because the culturally dominant stories of human suffering include psychiatric concepts and vernacular and a mental disorder may easily take the shape of an independent protagonist. In a similar way, the interpretations and expectations of help-seekers and their relatives may be largely shaped by the culturally dominant narratives. The power of (medicalized) narratives about distress, crisis, and suffering, thus, may be an important driver of psychiatrization, that can have decisive influence on how both top-level and bottom-level agents think, act, and decide.

## Concluding remarks

The ED as an area of contact between psychiatry and society appears to be a promising field for research on psychiatrization and on how it emerges from the institutions of mental healthcare. In the above cases and during the process of active interviewing, a wide array of drivers for top-down and bottom-up psychiatrization have surfaced. All these drivers may influence encounters in the ED in favor of psychiatric diagnosis and treatment, especially in cases where diagnosis is negotiable, because its clinical appropriateness is highly unclear. Besides, some soft and rather contextual factors that might promote psychiatrization in a more general way were identified, among which notions of normalcy or narratives about suffering circulating in society. From a broader perspective, the described cases and their analysis illustrate some fundamental difficulties that arise when certain human problems are understood, labeled, and treated as medical conditions. Even if the concept of psychiatrization were left aside, the case study may thus contribute to larger debates on the nature of mental illness, the use of offering explicitly *medical* interventions for those who experience mental distress for diverse reasons, and the appropriateness of diagnosis to capture the very meaning of these experiences.

In an attempt to summarize, some central findings of this case study about the ED as a place where psychiatrization potentially happens could be outlined as follows:

1. From a structural point of view, the ED can be characterized as a place where psychiatrists as top-level-agents directly interact with help-seekers as agents from the bottom-level. In more abstract terms, the ED, thus, constitutes an area of contact between individuals and the mental healthcare system or *between society and psychiatry*. Remaining in a spatial imagery, the ED may also be considered as a place from which psychiatric knowledge encroaches upon the social sphere.
2. Psychiatrization is about turning a phenomenon not (yet) psychiatric into something psychiatric. When a person in need encounters a psychiatrist for the very first time, the specific problems, or the conditions causal to coming to the ED, are not yet classified or interpreted. Therefore, some kind of gatekeeping is required here (Buchbinder, 2017). The ED is, thus, one of the special places where different kinds of personal issues or life problems, distress, or health conditions may be categorized as falling within psychiatric expertise—or not. It is precisely this fundamental openness of the situation in the ED which attracts scientific inquiry into the various reasons beyond clinical considerations why exactly certain cases are judged to be psychiatric and others are not (Dodier and Camus, 1998).
3. The ED is a typical place where psychiatric diagnoses are ascribed to individuals *for the very first time*. For research on psychiatrization, such places are of particular interest, since psychiatric diagnoses can be seen as the converging point of several sub-processes of psychiatrization (see Figure 1): They may be the entry point for service utilization, entail the prescription of psychotropics, be a result of the expansion of diagnostic categories, an act of pathologization of minor disturbances and a contribution to the high incidences of mental disorders.
4. With the ED being a “gateway to higher levels of medical care” (Grace, 2020, p. 876), the encounter in the ED is likely to be the starting point for some kind of treatment regime, ranging from direct admission to the psychiatric ward to the referral to out-patient services. Whichever psychiatric diagnosis is given, or whichever treatment is initiated, it may prompt gradual transformations in an individual's identity, over the course of which a person's narrative and sense of self may fundamentally change through the integration of psychiatric concepts (Rose, 2003; Martin, 2007; von Peter, 2013; Haslam and Kvaale, 2015). The ED may, thus, be the place where the psychiatric reshaping of identity as a central effect of psychiatrization *begins*.
5. From a social constructivist perspective and in the terms of Hacking, mental disorders *come into being* at the very moment, when a problem is interpreted through the psychiatric gaze and classified as belonging to a distinct diagnostic category (Hacking, 1985). In this regard, although the problems causal for a patient to come to the ED may have existed before, the ED may be one of the peculiar places where a mental disorder becomes real through diagnosis in an (at least) three-fold sense: (a) as a subjective conviction, which may gradually become a lived reality, (b) as an intersubjectively shared social reality, and (c) as a legal entity.

Further research could try to expand the inquiry into several directions. Some next steps to empirically establish psychiatrization in the ED and comparable settings could consist of (a) quantitatively expanding the scope of investigation through the inclusion of more cases from different hospitals, (b) shifting the focus of investigation by contrasting cases from the ED with cases from other settings where first contacts between psychiatry and society take place (e.g., the offices of general practitioners, crisis intervention teams, or community mental health services), (c) adding different perspectives on data collection and interpretation, e.g., by involving service-users or other professions than psychiatrists, (d) triangulating by the use of different methods (e.g., focus groups, expert interviews, participatory observation) and different types of evidence (e.g., patient records, discharge letters), and (e) gathering longitudinal data by following up the individual cases.

In particular, longitudinal data could generate new insights, especially when combined with a research design which features a control group (e.g., discharge with vs. discharge without psychiatric diagnosis). In this scenario, prospective research questions could be how the health status of individuals evolved after their ED contact, if and how individual problems or crises were settled, if other institutions of healthcare were consulted, and which other medical or non-medical actors stepped in when a person's indisposition was defined as being not primarily “psychiatric”. A longitudinal design would also allow inquiry into how the ED contact changed the identity and agency of the help-seekers or how the provided (psychiatric) explanations and concepts were incorporated or resisted in relationships, families, and other networks. Research of this kind could possibly also establish which specific interventions would be helpful to limit some of the negative aspects of psychiatrization, be it on the conceptual level through the promotion of alternative frameworks to understand mental distress or on the structural level through enabling professional counseling also in hospitals that has not been based on a psychiatric diagnosis for its reimbursement. Although hypothetical, conferring a psychiatric diagnosis might have been avoided in both described cases if there had been a chance for the psychiatrists in charge to practice such a simple measure as “watchful waiting” by scheduling a second appointment a few days later with Mr. A. and Ms. B (Iglesias-González et al., 2017). The cases thus may be hints that relatively obvious organizational constraints of healthcare institutions could be main targets for practical interventions to reduce the risk of psychiatrization in comparable gray area situations.

In addition, the above analysis indicates the need to continue theory development. The cases suggest that further theory building should attempt to clarify integral parts of the terminology with regard to the comprehensive model that served as starting point for this study. For instance, the diagnostic vagueness created by the classificatory systems was considered to be a *driver* of psychiatrization, while help-seekers' appeals to psychiatry for its non-medical functions were equally qualified as a driver. However, consulting psychiatry primarily for non-medical reasons may not be likely to result in psychiatrization, unless psychiatric diagnosis is applied in a space of vagueness. The vagueness of classificatory systems, thus, seems to function as an *enabler* of or *precondition* to other drivers of psychiatrization. This raises the question whether the concept of “drivers” is too broad and needs to be differentiated into separate categories.

Moreover, the classification of clinical psychiatrists as typical “top-level-agents” of psychiatrization may demand some modifications. Their actions may be shaped by top-level drivers, and their decisions may be guided by top-level knowledge, but the above material clearly shows that bottom-up drivers seem to exert a significant influence on how practitioners handle individual cases. Clinical psychiatrists working in the ED and comparable settings may, thus, quite often be in a mediating position between top- and bottom-level, a result, which also resonates recent studies on psychiatric emergency care (Lincoln, 2006; Buchbinder, 2017; Lane, 2020). Accordingly, a revised conceptualization of psychiatrization could possibly benefit from the introduction of an *intermediate category of agents*, which would serve to complexify the dichotomy between top-level and bottom-level agents.

## Data availability statement

The original contributions presented in the study are included in the article, further inquiries can be directed to the corresponding author.

## Author contributions

TB was responsible for devising the article, writing the manuscript, and drafting the final version.

## Funding

The open-access publication of this article was funded by the MHB Publication Fund supported by the German Research Association (DFG).

## Conflict of interest

The author declares that the research was conducted in the absence of any commercial or financial relationships that could be construed as a potential conflict of interest.

## Publisher's note

All claims expressed in this article are solely those of the authors and do not necessarily represent those of their affiliated organizations, or those of the publisher, the editors and the reviewers. Any product that may be evaluated in this article, or claim that may be made by its manufacturer, is not guaranteed or endorsed by the publisher.

## Footnotes

^1^As a matter of fact and somewhat ironically, the creation of DSM III was intended as an attempt to once and for all overcome the lack of reliability that characterized the psychoanalytically infused former two editions of the DSM by introducing an atheoretical, less etiological and more descriptive approach to psychiatric diagnosis (Horwitz, 2021). The historical background for this attempt was, among other factors, the harsh criticism of psychiatry originating from the social sciences and the anti-psychiatric movement and the loss of credibility of psychiatry as a scientifically based medical specialty, propelled by its inability to tell the mentally ‘sane' from the ‘insane' as famously unveiled by the Rosenhan experiment (Rosenhan, 1973).

^2^The power of classificatory systems may also be underlined by the very rare examples of de-psychiatrization through eliminating certain categories, as happened to homosexuality (Drescher, 2015) and seems to be currently happening with the transformation of *gender identity disorder* from ICD-10 into *gender incongruence* in ICD-11 (Reed et al., 2016).

^3^It is worth noting that following the most radical critics of psychiatry, this distinction would be entirely meaningless, as there just are no people being rightly classified as mentally ill, when the idea of the existence of mental disorders as a whole is refused.

^4^Interestingly, the ICD-10 acknowledges that also non-medical conditions may lead to the use of medical services and provides the so-called “Z-codes” to code for the different reasons for these encounters. Z-codes are intended to mark individual social or economic needs that may not require a specific medical intervention but were causal for the medical consultation anyway and might be relevant as context for future treatments. However, documentation rates for Z-codes are very low, as consultations based only on Z-codes are usually not re-imbursed by health insurances (McCormack and Madlock-Brown, 2021). For the same reason, it is very unlikely that Z-codes have the potential to replace psychiatric diagnosis using F-codes in healthcare settings, although they might often be the more accurate description for the reason for a medical consultation.
